# Time-Resolved Fluorescent Immunochromatography of Aflatoxin B1 in Soybean Sauce: A Rapid and Sensitive Quantitative Analysis

**DOI:** 10.3390/s16071094

**Published:** 2016-07-14

**Authors:** Du Wang, Zhaowei Zhang, Peiwu Li, Qi Zhang, Wen Zhang

**Affiliations:** 1 Oil Crops Research Institute of the Chinese Academy of Agricultural Sciences, Wuhan 430062, China; wang416929@gmail.com (D.W.); zhangwen@oilcrops.cn (W.Z.); 2 Key Laboratory of Biology and Genetic Improvement of Oil Crops, Ministry of Agriculture, Wuhan 430062, China; 3 Key Laboratory of Detection for Mycotoxins, Ministry of Agriculture, Wuhan 430062, China; 4 Laboratory of Risk Assessment for Oilseeds Products (Wuhan), Ministry of Agriculture, Wuhan 430062, China; 5 Quality Inspection and Test Center for Oilseeds Products, Ministry of Agriculture, Wuhan 430062, China

**Keywords:** time-resolved fluorescent immunochromatographic strip, aflatoxin B1, soybean sauce, food safety

## Abstract

Rapid and quantitative sensing of aflatoxin B1 with high sensitivity and specificity has drawn increased attention of studies investigating soybean sauce. A sensitive and rapid quantitative immunochromatographic sensing method was developed for the detection of aflatoxin B1 based on time-resolved fluorescence. It combines the advantages of time-resolved fluorescent sensing and immunochromatography. The dynamic range of a competitive and portable immunoassay was 0.3–10.0 µg·kg^−1^, with a limit of detection (LOD) of 0.1 µg·kg^−1^ and recoveries of 87.2%–114.3%, within 10 min. The results showed good correlation (R^2^ > 0.99) between time-resolved fluorescent immunochromatographic strip test and high performance liquid chromatography (HPLC). Soybean sauce samples analyzed using time-resolved fluorescent immunochromatographic strip test revealed that 64.2% of samples contained aflatoxin B1 at levels ranging from 0.31 to 12.5 µg·kg^−1^. The strip test is a rapid, sensitive, quantitative, and cost-effective on-site screening technique in food safety analysis.

## 1. Introduction

Aflatoxins are a group of extremely toxic metabolites produced by Aspergillus flavus and A. parasiticus, which grow on foods [1]. Although more than 20 aflatoxins have been identified, the major aflatoxins of concern are designated aflatoxins B1 (AFB1), B2, G1 and G2 [2,3]. According to the International Agency for Research on Cancer, AFB1 are listed in group I as human carcinogens [4], because of their highly hepatotoxic, teratogenic, mutagenic, and carcinogenic effects [5].

Soybean sauce is a widely consumed condiment worldwide and used extensively as a seasoning due to its unique taste and aroma [6,7]. It is usually derived from soybeans that are mixed with roasted grain (usually wheat, rice or barley) and fermented for several months. The source of soybean sauce is easily contaminated with aflatoxins. Most countries have set up regulations for the maximum permissible levels of AFB1 residues in soybean sauce, such as 2.0 µg·kg^−1^ in the European Union and 5.0 µg·kg^−1^ in China [8,9].

Rapid, quantitative, low-cost, and reliable tests for AFB1 detection in soybean sauce are urgently needed. Generally, AFB1 is detected via high performance liquid chromatography (HPLC), ultrahigh performance liquid chromatography tandem mass spectrometry (UHPLC-MS/MS), enzyme-linked immunosorbent assay (ELISA) and membrane matrix immunoassays [10,11,12]. Instrumental methods provide accuracy, precision, sensitivity, and reproducibility. However, they are inappropriate for rapid screening due to the need for tedious sample preparation, expensive equipment, and highly skilled operators. On the other hand, immunosensing represents a promising alternative [13,14,15]. Immunosensing is a new, rapid and on-site screening tool with several advantages, such as simplicity, rapid and immediate results, low cost, without the need for skilled technicians or expensive equipment.

Quantitative fluorescent immunosensing methods have been developed recently for the analysis of aflatoxins. They include fluorescence polarization immunoassay and time-resolvedfluorescence immunoassay (TRFIA). Liu et al. reported an extract-free immunochromatographic assay based on fluorescent microsphere probes for qualitative AFB1 detection in soybean sauce, resulting in a visible LOD of 2.5 mg/L [16]. However, no report of quantitative AFB1 detection in soybean sauce is available. The introduction of TRFIA in 1980s offered advantages such as rapidity, sensitivity, convenience, and potential for both quantitative and qualitative sensing [17,18,19]. Nevertheless, a major concern related to the inherently low fluorescence of Eu(III), which was difficult to measure. In aqueous phase, especially, the smaller molar extinction coefficients and low quantum yield hampered its sensitivity [20,21]. Doping Eu(III) in nanosphere capsules (Eu-nanospheres) for signal amplification dramatically enhanced the sensitivity thousands of times [22,23,24,25,26].

We have developed a competitive and quantitative immunoassay in which Eu-nanospheres were conjugated with antibodies against AFB1, serving as the immunoreagent. The portable assay was used to record the time-resolved fluorescence. The limit of detection (LOD), dynamic range, and recovery were recorded. This proposed method facilitates rapid, sensitive and extensive quantification of AFB1 and other toxins in soybean sauce.

## 2. Materials and Methods

### 2.1. Reagents and Apparatus

AFB1 standard solutions, AFB_2a_-BSA and rabbit anti-mouse IgG were purchased from Sigma-Aldrich (St. Louis, MO, USA). Mannitol, sucrose, bovine serum albumin (BSA) 1-ethyl-3-(3-dimethylaminopropyl) carbodiimide (EDC) and polyvinyl pyrrolidone (PVP) were obtained from Roche Applied Science (Indianapolis, IN, USA). The nanospheres enclosing fluorescent europium (Eu-nanospheres) measuring 190 nm in diameter and 1.02% solid content were supplied by Shanghai Uni Bio-tech Company Limited (Shanghai, China). The 95 NC membranes were purchased from Millipore Corporation (Bedford, MA, USA). All aqueous solutions were prepared in ultra-pure water of 18.2 MΩ purified using a Milli-Q quality water system (Bedford, MA, USA). All chemicals were of analytical grade unless otherwise stated.

The antibody (Anti-AFB1 mAb3G1) was produced in our laboratory and further purified with a protein G immunoaffinity column [22,23]. The antibody AFB1_3G1_ showed high specificity to aflatoxin B1, with the following cross-reactivities to aflatoxin B1, B2, G1 and G2: 100%, 6.4%, <0.02% and <0.02%, respectively. The sensitivity (expressed by IC50) of MAb 3G1 to aflatoxin B1 was 1.6 ng/mL. Soybean sauce samples were purchased from the local market in Wuhan, China.

The portable time resolved fluorescent immunochromatographic (TRFIA) apparatus was homemade. The signal was detected under the exciting light at a wavelength of 365 ± 10 nm, and signal was acquired at 613 ± 10 nm using the photomultiplier tube (PMT) [22]. A typical delay of 400 μs occurred when the emission light was collected from the excited light source. After immunochromatographic reaction, the test line (T line) and control line (C line) peak areas were recorded and calculated using data processing software for quantitative analysis. The T line was used to detect the AFB1.

### 2.2. Conjugation of Eu-Nanospheres to Antibody

EDC was used to develop active nanospheres with carboxyl groups in aqueous media. EDC is the most effective reagent used to link amino and carboxyl groups. The probe was synthesized according to the published method with slight modifications [24,26,27]. First, 1 mg/mL of antibody was dispersed in phosphate buffer (0.2 mmol·L^−1^, pH 7.2). Next, 200 μL of Eu-nanospheres was added to 800 μL of boric acid buffer and ultrasonicated (30 kHz frequency, 20% amplitude, 0.6 intermittent frequencies) at 20 °C for 2 min. Then, 40 μL of EDC (15 mg/mL) was added and stirred for 15 min, followed by centrifugation at 17,000 g for 10 min. The precipitate was re-suspended in 1.0 mL of boric acid buffer by ultrasonication. A gradient of antibody concentrations was added, and the solution was vortexed for 12 h before centrifugation at 17,000 g for 10 min. Subsequently, the supernatant was obtained for protein quantification. The residue was re-suspended in 1.0 mL of boric acid buffer (containing 0.5% BSA), and vortexed for 2 h. The solutions were diluted and lyophilized in the penicillin bottle, and stored at 4 °C before use. The pH of the buffer solutions and the antibody dosage were optimized before the next step.

### 2.3. Preparation of the Immunochromatographic Strip

The immunochromatographic (IC) strip contained an NC membrane, plastic backing plate, sample pad, and absorbent pad. The IC was prepared as previously described [28]. The sample pad was treated with a buffer blocked and dried at 37 °C for 12 h. The AFB_2a_-BSA and rabbit anti-mouse IgG were coated on NC membrane as T line and C line, respectively, using Bio Dot XYZ Platform at an appropriate jet rate. The distance between the T line and the C line was 10 mm. The NC membrane, sample pad and absorbent pad were laminated and pasted onto a plastic backing plate. The whole assembly of scale board was cut longitudinally and divided into strips using a guillotine cutter (CM 4000) measuring 4.0 mm × 70 mm and stored at 4 °C.

### 2.4. Evaluation of TRFIA

An indirect competitive immunoassay was performed using the IC strip. The Eu- probe was diluted 50, 100, 200, 300 and 500 times with protective reagents (containing 6% sucrose, 4% BSA, 1% mannitol dilution, and 1% PVP). The optimal Eu-probe concentration was similarly screened as a checkerboard titration in an ELISA using the IC strip. AFB1-free samples were analyzed during the development of the technique as well as in the validation study to verify the stand curve, the LOD, limit of quantitation (LOQ), recovery, and repeatability.

AFB1-free sample extracts spiked with AFB1 standard solutions of 0.1, 0.3, 1.5, 6.0, 9.0, and 12.0 µg·kg^−1^, were analyzed with the IC strip test to estimate the sensitivity and linear range of the method, respectively. The fluorescent intensities from the T and C lines were recorded as (T) and (C), respectively, using portable TRFIA. The ratio of the value (T) to the value (C) was calculated as the signal ratio (Y). A calibration curve was fitted and expressed as Y versus the natural log of the AFB1 concentration (C_AFB1_) [27]:

Y (value T/C) = a·X (ln C_AFB1_) + b

The LOD was calculated from the 20 AFB1-free samples using standard curves. The LOQ was calculated as 3 times the LOD. All the experiments were repeated five times.

The recovery was studied to evaluate the precision of TRFIA, AFB1 standard solutions were added to fortified AFB1-free samples at five levels prior to the extraction step. The spiked samples were analyzed using the method described above.

The inter-batch and intra-batch variation was studied to evaluate the repeatability. Each assay was repeated 5 times. The inter-batch was determined from 5 batches, within which a mean value was recorded from 5 strips within the same batch. The coefficient of variation (CV) was evaluated for repeatability. The TRFIA method was validated by comparing the results of TRFIA and HPLC methods.

### 2.5. Sample Treatment for TRFIA

A representative 10 mL sample placed in a 50 mL centrifuge tube and extracted with 20 mL of 70% methanol (*v/v*) was vortexed for 30 s, and the extract was homogenized for 2 min. Subsequently, the solution was centrifuged at 8000 g for 2 min. The supernatant (1.0 mL) was diluted with 3.0 mL buffer before measurement using TRFIA. After mild vortexing, 150 μL solution was dropped into the penicillin bottle. The IC strip was inserted into the penicillin bottle and incubated at 37 °C for 6 min. The components of soybean sauce were more complex, and included salts, different amino acids, and pigments. Therefore, appropriate dilution ratio and diluted buffers were needed to reduce matrix interference.

### 2.6. Sample Treatment for HPLC Analysis

The TRFIA was validated based on a published analytical method used to extract and purify the AFB1 in soybean sauce. The sample was purified on immunoaffinity column. One milliliter aliquot in methanol was injected into an HPLC equipped with fluorescence detector (λex = 365 nm, λem = 440 nm). The analytical column comprised an Alltima C18 (150 × 4.6, particle size 5 µm, Alltech, Grace, IL, USA). The mobile phase consisted of a mixture of HPLC grade methanol-water (55:45) eluted at a flow rate of 1.0 mL·min^−1^. The temperature of the column was 25 °C and the injection volume of suspended sample was 10 µL [29,30].

## 3. Results and Discussion

### 3.1. Optimization of Eu-Probe

#### 3.1.1. Optimal pH of Boric Acid Buffer Solution for Conjugation

To establish the optimal reaction system, a boric acid buffer solution at different pH values of 7.0, 7.4, 7.8, 8.2, 8.7, and 9.0, was used, respectively. The prepared Eu-probe was dissolved in boric acid buffer and added to strips to detect the signal on T line using the portable TRFIA. The result showed that excessively high or low pH promoted conjugation coagulation and obliterated the color of T line under UV light. The optimal pH was 8.2. The result was close to the AFB1-antibody isoelectric point, as reported previously [26,28].

#### 3.1.2. Optimal Antibody Level Labeled with Eu-Nanospheres

The Eu-probe was prepared with different mass ratios of the antibody and Eu-nanospheres (0, 10, 20, 30, 40, 50, and 60 ng) in boric acid buffer. First, the lowest stabilizing concentration of antibody prevented coagulation. Following conjugation, the supernatant was collected for protein quantification for determination of protein absorbance. The results suggested that the absorbance increased at an antibody level exceeding 30 ng and the Eu-nanospheres conjugated with antibody were less than 30 ng. Further, the antibody concentration less than 30 ng showed low fluorescent intensity on T line at the same dilution. The blank solution and 0.5 µg·kg^−1^ AFB1 standard solution were evaluated for antibody digestion during the coupling of antibody and Eu-nanospheres. The result showed that at 30 ng, the antibodies showed better CVs on T line, along with better stability and sensibility.

### 3.2. Optimization of the IC Strip

**Figure 1:**
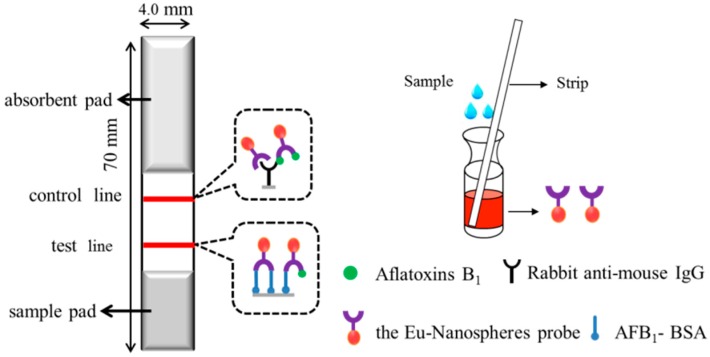
Schematic outlining the principle of competitive assay in immunochromatographic (IC) strip format.

The IC strip is a single-step test based on a competitive immunoassay format (Figure 1). The concentrations of AFB_2a_-BSA and rabbit anti-mouse IgG were prepared with serial dilutions of 0.1 to 1.0 mg·mL^−1^. The optimal concentrations were similarly screened as a checkerboard titration in an ELISA. Inhibitory rates were recorded to determine theoptimum concentrations. It was found that 0.8 µL·cm^−1^ AFB_2a_-BSA (0.25 mg·mL^−1^) and 0.7 µL·cm^−1^ rabbit anti-mouse IgG (0.5 mg·mL^−1^) were optimized to dispense on the T line and C line, respectively.

The sample pad was used to reduce matrix interference. The solutions containing 1.0% BSA, 2.5% sucrose, 2.5% Tween 20, and 0.02% NaN3 in 0.01 mol/L PBS (pH 7.4),were used to block the sample pad, which guaranteed a clear redline on the test line under the UV light for negative samples [27].

### 3.3. Optimization of TRFIA

Based on the competitive theory, the extract solutions or AFB1 standard solutions were pipetted into the penicillin bottle. After mild vortexing, the IC strip was inserted into the penicillin bottle and incubated at 37 °C for 6 min.

**Figure 2:**
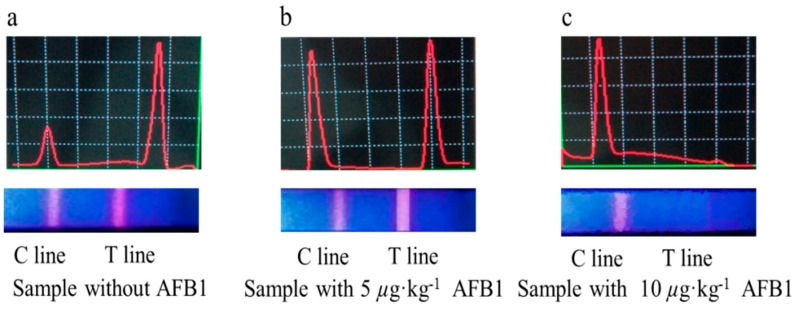
Different concentrations of AFB1 under UV light and portable TRFIA ((**a**) sample without AFB1; (**b**) sample with 5 µg·kg^−1^ AFB1; (**c**) sample with 10 µg·kg^−1^ AFB1).

In the negative samples (fortified AFB1-free), the Eu-probe flowed laterally along the strip toward T line. The Eu-probe was captured by the immobilized AFB2a-BSA on T line. Excess Eu-probe was continuously captured by the rabbit anti-mouse IgG on the C line, as seen in Figure 2a.

In the positive sample, the AFB1 toxins conjugated with Eu-probe, the compound did not interact with the immobilized AFB_2a_-BSA on the T line, but reached the C line. The surplus Eu-probe flow was laterally captured by the T line as indicated by a faint red color (i.e., a positive sample with 5 µg·kg^−1^ AFB1, Figure 2b) or no color (i.e., a positive sample with 10 µg·kg^−1^ AFB1), as seen in Figure 2b,c.

**Figure 3:**
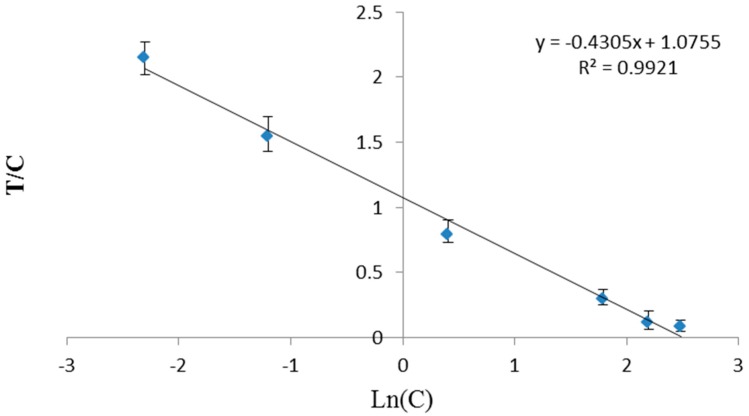
Standard curve by of soybean sauce sample.

To establish a standard curve, the AFB1-spiked sample was employed. The ratio of T line value to C line value versus the natural logarithm of the concentration (Ln c), yielded good correlation (R^2^ > 0.99) (Figure 3). The concentration 10.0 µg·kg^−1^ in the strip showed no color under UV light, as seen in Figure 2c.

For the LOD, the mean value of 5 repeated experiments was recorded in 20 blank soybean bean samples. Based on the sample standard curve, the AFB1 concentration was further calculated and defined as the LOD. The LOD was 0.1 µg·kg^−1^ for the soybean sauce matrices, LOQ was 0.3 µg·kg^−1^, at the wider dynamic ranges of 0.3–10.0 µg·kg^−1^, respectively. The proposed method showed a lower LOD compared with the ELISA, gold particlesstrip and the reported previously using fluorescent microsphere probe-based IC strip more than 3 times [16,31]. More importantly, the portable apparatus allowed quantitative analysis in addition to the qualitative result visible to the naked eye.

In the recovery experiment, different soybean sauce samples were used in the inter batch repeated 5 times. Results showed an average recovery of 111.0%, ranging between 87.2% and 114.3%, with mean CVs of 6.0% (3.4%–8.6%), as shown in Table 1. To accurately determine AFB1 at high concentrations over 10.0 µg·kg^−1^, the soybean bean sample was diluted using 20% methanol.

To test the repeatability, random soybean sauce samples were determined by HPLC before use. As shown in Table 2, the intra-batch repeatability revealed low CVs of 4.6%–9.1% while the intra-batch repeatability ranged from 5.2% to 9.4%, suggesting that the method was reproducible and addressed the regulation of AFB1 in soybean sauce samples.

### 3.4. Optimization of Sample for TRFIA

The 70% methanol solutions were used to extract the AFB1 in samples. Previous reports [26] showed that high organic solvents improve the extraction efficiency. However, they reduced the antibody activity in the NC membrane. Thus, the extraction solution should be diluted to reduce the methanol concentration. The result demonstrated that a buffer dilution of 1:3 improves the signal intensity of the T line. The buffer solutions containing 0.5% PVP in 0.01 mol/L of PBS (pH 7.4) improved reaction efficiency and reduced the CV.

### 3.5. Validation by HPLC

A total of 53 soybean sauce samples were analyzed by TRFIA, and 34 of these samples (64.2% of 53 samples) were contaminated by AFB1. The average content was 0.81 µg·kg^−1^, and the AFB1 concentration ranged from 0.31 to 12.5 µg·kg^−1^. Only 3 samples exceeded AFB1 concentration over 5.0 µg·kg^−1^. In order to verify the accuracy of TRFIA, we randomly selected 10 samples containing the 3 positive samples for accuracy. Therefore, as shown in Table 3, the results of TRFIA were consistent with the results of HPLC, the HPLC method referred followedas Chinese National Standard (2003) [30]. TRFIA increased the efficiency of large-scale evaluation of soybean sauce on-site and facilitated rapid screening of samples containing high concentrations of AFB1.

## 4. Conclusions

A TRFIA was developed for rapid and quantitative detection of AFB1 in soybean sauce samples. Nanospheres enclosing fluorescent europium before conjugation with anti-AFB1 antibody via EDC dramatically enhanced the sensitivity. The TRFIA enabled the detection of AFB1 in soybean within 10 min, with an LOD of 0.1 µg·kg^−1^, considerable linear range of 0.3–10.0 µg·kg^−1^, and recovery of 87.2%–114.3%. The CVs ranged from 3.4% to 8.6%. Furthermore, the intra-batch and inter-batch comparison revealed excellent repeatability and reproducibility. A high concordance of TRFIA and HPLC methods was recorded in soybean sauce samples. The TRFIA method can be widely utilized in rapid on-site analysis of AFB1 in soybean sauce samples.

## Figures and Tables

**Table 1:** The recovery and coefficients of variation (CVs) of time resolved fluorescent immunochromatographic (TRFIA) method.

Sample	Number	Spike µg·kg^−1^	Result by TRFIA µg·kg^−1^						CV%	Recovery %
			1	2	3	4	5	Mean		
Soybean sauce	1	ND	ND	ND	ND	ND	ND	-	-	-
	2	0.80	0.99	0.97	0.90	0.79	0.92	0.91	8.6	114.3
	3	4.00	4.57	3.88	3.69	4.33	4.22	4.14	8.5	103.5
	4	8.00	7.90	7.39	7.74	8.43	7.26	7.75	6.0	96.8
	5	12.00	11.45	11.98	13.01	12.89	12.87	12.44	5.7	103.7
Soybean sauce	1	ND	ND	ND	ND	ND	ND	-	-	-
	2	2.00	2.10	2.07	1.93	2.20	2.29	2.12	6.4	105.9
	3	5.00	5.02	5.24	5.46	5.34	5.09	5.23	3.4	104.6
	4	10.00	9.42	9.89	9.65	10.37	10.57	9.98	4.5	99.8
	5	15.00	12.98	12.07	13.58	13.12	13.68	13.09	5.0	87.2
Average									6.0	110.0

ND: “not detected”.

**Table 2:** Intra- and inter- batch CV tests ofin TRFIA sensing.

Soybean Sauce Sample	HPLC Detected AFB_1_ µg·kg^−1^	Intra-Batch (n = 5)		Inter-Batch (n = 5)	
		Average Detected Value µg·kg^−1^	Average CV %	Average Detected Value µg·kg^−1^	Average CV %
1#	ND	ND	-	ND	-
2#	0.8	0.69	7.9	0.68	8.7
3#	3.2	3.43	4.6	3.58	5.2
4#	8.9	9.30	5.3	9.20	5.6
5#	ND	ND	-	ND	-
6#	0.3	0.32	8.9	0.27	9.2
7#	4.7	4.43	6.6	3.98	5.9
8#	12.4	13.2	5.3	13.7	5.6
9#	6.4	6.00	6.4	5.80	7.2
10#	0.8	0.69	9.1	0.68	9.4

ND: “not detected”.

**Table 3:** AFB1 analysis: TRFIA vs. HPLC methods.

Number	AFB_1_ Result by HPLC µg·kg^−1^	AFB1 Result by TRFIA µg·kg^−1^
1#	5.20	4.90
2#	6.40	6.70
3#	9.80	10.7
4#	3.20	2.75
5#	2.90	2.45
6#	0.80	0.70
7#	1.30	1.50
8#	3.60	4.20
9#	0.31	0.44
10#	3.60	3.80
